# PREOPERATIVE MANOMETRY FOR THE SELECTION OF OBESE PEOPLE CANDIDATE TO SLEEVE GASTRECTOMY

**DOI:** 10.1590/0102-6720201700030013

**Published:** 2017

**Authors:** Antonio Carlos VALEZI, Fernando Augusto HERBELLA, Jorge MALI-JUNIOR, Mariano de Almeida MENEZES, Mário LIBERATTI, Rafael Onuki SATO

**Affiliations:** 1 Digestive System Surgery, Department of Surgery, State University of Londrina, Londrina, PR, Brazil

**Keywords:** Obesity, Manometry, Esophagogastric junction

## Abstract

**Background:**

Sleeve gastrectomy may alter esophageal motility and lower esophageal sphincter pressure.

**Aim:**

To detect manometric changings in the esophagus and lower esophageal sphincter before and after sleeve gastrectomy in order to select patients who could develop postoperative esophageal motilitity disorders and lower esophageal sphincter pressure modifications.

**Methods:**

Seventy-three patients were selected. All were submitted to manometry before the operation and one year after. The variables analyzed were: resting pressure of the lower esophageal sphincter, contraction wave amplitude, duration of contraction waves, and esophageal peristalsis. Data were compared before and after surgery and to the healthy and non-obese control group. Exclusion criteria were: previous gastric surgery, reflux symptoms or endoscopic findings of reflux or hiatal hernia, diabetes and use of medications that could affect esophageal or lower esophageal sphincter motility.

**Results:**

49% of the patients presented preoperative manometric alterations: lower esophageal sphincter hypertonia in 47%, lower esophageal sphincter hypotonia in 22% and increase in contraction wave amplitude in 31%. One year after surgery, manometry was altered in 85% of patients: lower esophageal sphincter hypertonia in 11%, lower esophageal sphincter hypotonia in 52%, increase in contraction wave amplitude in 27% and 10% with alteration in esophageal peristalsis. Comparing the results between the preoperative and postoperative periods, was found statistical significance for the variables of the lower esophageal sphincter, amplitude of contraction waves and peristalsis.

**Conclusion:**

Manometry in the preoperative period of sleeve gastrectomy is not an exam to select candidates to this technique.

## RESUMO

**Racional:**

A gastrectomia vertical pode determinar alterações na motilidade esofágica e no esfíncter inferior do esôfago.

**Objetivo:**

Estudar as alterações manométricas do esfíncter inferior do esôfago e do esôfago antes e depois da operação a fim de selecionar pacientes que pudessem desenvolver alterações pós-operatórias.

**Métodos:**

Setenta e três pacientes foram selecionados. Todos foram submetidos à manometria antes da operação e um ano após. As variáveis analisadas foram: pressão do esfíncter inferior do esôfago, amplitude e duração das ondas de contração e peristaltismo esofágico. Os dados foram comparados entre si antes e depois da operação e também com grupo controle saudável e não obeso. Critérios de exclusão foram: operação gástrica prévia, história de refluxo ou achado endoscópico de esofagite de refluxo ou de hérnia de hiato, diabete e uso de medicamentos que pudessem afetar a motilidade do esôfago ou do esfíncter esofágico inferior.

**Resultados:**

49% dos pacientes apresentaram alterações no pré-operatório: hipertonia do esfíncter em 47%, hipotonia do esfíncter em 22% e aumento na amplitude das ondas de contração em 31%. Um ano após, a manometria encontrou-se alterada em 85% dos pacientes: hipertonia do esfíncter em 11%, hipotonia do esfíncter em 52%, aumento na amplitude das ondas de contração em 27% e 10% com alteração no peristaltismo esofágico. Comparando-se os resultados entre o pré e pós-operatório encontrou-se significância estatística para a pressão do esfíncter inferior do esôfago, amplitude das ondas de contração e peristaltismo.

**Conclusão:**

A manometria no pré-operatório da gastrectomia vertical não é fator de seleção dos candidatos a essa técnica.

## INTRODUCTION

Obesity is associated with an increased incidence of gastroesophageal reflux (GER)^9^. Weight loss determined by bariatric surgery can reduce these symptoms. The improvement depends on the surgical technique employed, for example, the adjustable gastric band, in spite of inducing weight loss, may worsen GER; gastric bypass decreases weight and shows excellent results on the improvement of the GER symptoms^21^ ^,^ ^32^. Vertical gastrectomy is a good option for weight loss, but transforming the stomach into a cylindrical structure and altering the anatomy of the esophagogastric junction, it may alter the function of the lower esophageal sphincter (LES) and, consequently, some patients submitted to this technique may develop GER^5^ ^,^ ^6^ ^,^ ^12^. Several studies have studied the symptoms of GER in the postoperative period of vertical gastrectomy, but few have evaluated the esophagogastric junction^3^ ^,^ ^16^.

The purpose of this study was to determine the manometric changes of the LES and esophageal body before and after performing vertical gastrectomy compared to healthy volunteers. The hypothesis was that surgical manipulation near the esophagogastric angle during the operation could affect LES function and esophageal motility.

## METHODS

This study was approved by the ethics and research committee of the State University of Londrina. It is a prospective cohort with a consecutive sample of the convenience of 87 obese subjects submitted to vertical gastrectomy from April 2012 to March 2014. The surgical indication obeyed the international criteria for performing bariatric surgery. Exclusion criteria were: previous gastric surgery, history of GERD or endoscopic finding of reflux esophagitis or hiatal hernia, diabetes and use of medications that could affect esophageal or lower esophageal sphincter motility. Patients who had postoperative complications that required surgical or endoscopic treatment, those who did not complete the study or those who refused to participate in the study were excluded from the analysis, so the data refer to 73 patients: 18 men and 55 women, with a mean age of 40.2 years (19-61) and mean body mass index (BMI) of 41.1 kg / m^2^ (35-46).

The patients selected for the study were submitted to manometry before and one year after surgery. The variables analyzed were resting pressure of the LES in mmHg (considering normal values ​​between 10-35 mmHg), contraction wave amplitude in mmHg (normal values ​​of 64-154 mmHg), duration of contraction waves in seconds and esophageal peristalsis. The data were compared before and after the operation and the findings of the healthy and non-obese control group, composed of 10 volunteers, were also compared. The control group did not present gastrointestinal symptoms, previous abdominal operations and did not use any type of medication that could interfere in the esophageal motility or the LES pressure. All patients were operated by the same surgeon and the manometry performed by a single examiner. All obese participants in the study signed informed consent for the study.

### Esophageal manometry

The test was performed with an eight-channel, water-infused device after an 8 h fast. The manometric data were obtained through 10 swallows of 5 ml of water with interval of 5 min. Synetics^R^ software, USA, was used for data interpretation and analysis. Ten days before the study, drugs that could interfere with esophageal motility and proton pump inhibitors were discontinued. There were no complications during the exams.

### Surgical technique

Vertical gastrectomy was performed with the surgeon between the patient’s legs. Initially, the vessels were released from the great gastric curvature from 4 cm of the pylorus until the gastric fundus was completely released and the diaphragmatic pillar was seen. After this surgical time a 32 F oro-gastric probe was introduced to the duodenum. The stomach was sectioned using a laparoscopic stapler along the gauge probe, starting 4 cm from the pylorus and continuing cranially to the esophagogastric angle. The last stapling was done 1 cm laterally to the esophagus. After removal of the stomach from the abdominal cavity, invaginating continuous suture was made with absorbable yarn of the staple line and methylene blue test was done. There was no intraoperative complication or need for conversion to laparotomy.

### Statistical analysis

The variables were analyzed using non-parametric tests, Wilcoxon test for paired samples and Mann-Whitney for simple samples. Statistical significance was considered when *p* was less than or equal to 0.05.

## RESULTS

Of the 73 patients, 36 (49%) presented manometric changes in the preoperative period. The alterations found in this group were: LES hypertonia in 17 (47%), eight LES hypotonia (22%) and 11 with an increase in contraction wave amplitude (31%). One year after the operation, manometry was altered in 62 patients (85%). The findings in this group were: LES hypertonia in seven (11%), 32 LES hypotonia (52%), 17 with an increase in contraction waves amplitude (27%) and six (10%) with alterations in esophageal peristalsis The control group did not present manometric alterations (Table 1).

**TABLE 1:** Manometric findings (n=73)

Findings	Pre	Post	Control
LES hypertonia	17	7	-
LES hypotonia	8	32	-
High contratility	11	17	-
Disperistalsis	*-*	6	-

LES= lower esophageal sphincter; pre=preoperative; post=postoperative

Lower esophageal sphincter pressure before and after the operation was 26.5 9.1 mmHg and 12.6±8.7 mmHg, respectively. The amplitude of the contraction waves was 133.1±38.7 mmHg in the preoperative period and in the postoperative period was 146.5±37.7 mmHg. The duration of pre and postoperative contraction waves was 4.2±1.1 s and 5.7±1.1 s, respectively. Normal peristalsis occurred in 100% of the patients before the operation and in 90% after (Table 2).

**TABLE 2:** Manometric changes

	Pre	Post	Control
LES pressure (mmHg)	26.5 ± 9.1	12.6 ± 8.7	17.8 ± 4.9
Wave amplitude (mmHg)	133.1 ± 38.7	146.5 ± 37.7	100.3 ± 30.1
Wave duration (mmHg)	4.2 ± 1.1	5.7 ± 1.1	5.0 ± 0.9
Normal peristalsis (%)	100	90	100

LES= lower esophageal sphincter; pre=preoperative; post=postoperative

Comparing the results of the preoperative period with the control group, statistical significance was not found for the variables analyzed. When comparing the postoperative results with the control, there was a significant difference in the LES pressure and the amplitude of the contraction waves. Comparing the results between the preoperative and postoperative periods, statistical significance was found for the variables LES pressure, amplitude of contraction waves and peristalsis (Table 3).

**TABLE 3:** Statistical analysis of pre and postoperative findings

	Medians			p		
	Pre	Post	Control	Pre x Post	Pre x Control	Post x Control
LES pressure (mmHg)	26.5	12.6	17.8	0.0001	0.2657	0.0001
Wave amplitude (mmHg)	133	146.5	100.3	0.0001	0.5789	0.0001
Wave duration (mmHg)	4.2	5.7	5.0	0.4387	0.6769	0.8243
Normal peristalsis (%)	100	90	100	0.0001	0.6239	0.0001

LES= lower esophageal sphincter; pre=preoperative; post=postoperative

## DISCUSSION

In this research we studied patients without GER because the objective was to evaluate the effects of vertical gastrectomy on esophageal motility and lower esophageal sphincter function. The absence of GER was defined by the absence of symptoms and normal endoscopic examination. The lack of esophageal phmetry in this population may be a bias in this research, since GER may be present in asymptomatic patients^7^.

A control group was constituted in this research, due to the fact that manometric changes in the esophagus can occur in non-obese asymptomatic individuals^13^ ^,^ ^14^ ^,^ ^24^ ^,^ ^25^.

Manometry is an important tool for the study of LES and esophageal body function^2^. High-definition manometry is a better examination in this respect, but it is not available in our service, so conventional manometry was used^11^. The resting pressure of the LES was used because it represents its isolated pressure, without the interference of the diaphragmatic pillars^2^.

In the studied population, we found preoperative manometric changes in 49% of the obese, with an increase in the amplitude of the contraction waves. A previous study by our team^30^ identified changes in manometry in 45.6% of the obese studied and the amplitude of the contraction waves was also predominant.

Several publications have shown that vertical gastrectomy is an effective method for weight loss and improvement of comorbidities^1^ ^,^ ^17^ ^,^ ^31^; however, its effect on the possibility of GER is controversial^18^ ^,^ ^20^ ^,^ ^26^ ^,^ ^27^. Mechanisms that may lead to GER after vertical gastrectomy include increased intragastric pressure, modification of the gastroesophageal junction and alteration in the mechanics of LES^19^ ^,^ ^23^.

Manometric studies after vertical gastrectomy showed a significant decrease in LES pressure^4^ ^,^ ^8^ ^,^ ^10^. In this study, there was alteration of the esophageal function, with an increase in the contraction wave amplitude and worsening of the peristalsis after the operation, and there was also a significant decrease in the LES pressure.

The worsening of esophageal motor function is most likely due to the increase in the pressure of the interior of the stomach after vertical gastrectomy and the decrease of the pressure of the LES possibly occurs due to the injury of the muscular fibers of the esophagogastric transition^28^ ^,^ ^29^.

Data on manometric changes of the LES are controversial. Braghetto et al.^3^ observed a decrease in the resting pressure of the LES six months after vertical gastrectomy, probably due to the lesion of the arched fibers of the cardia. In this study, we found similar data, with a significant decrease in LES pressure.

Kleidi et al.^15^ assessed asymptomatic patients regarding GER and with normal LES pressure in the preoperative period and found an increase in the extent of the LES; however, they observed a decrease in contraction of the distal esophagus that could interfere with esophageal whitening, determining GER independently of LES pressure. Petersen et al.^22^ found an increase in LES pressure shortly after the operation, and that this finding was not dependent on weight loss, but on placement of the stapler further away from the esophagus to avoid lesion of the sphincter fibers.

Gastroesophageal reflux after vertical gastrectomy can vary from 2.8 to 13%^4^ ^,^ ^28^ ^,^ ^29^. Manometry should be routine preoperative examination in obese candidates for this surgical technique, because if the patients present low pressure of the LES, vertical gastrectomy should not be performed^32^.

This is also the opinion of the authors of this study and if we were able to select preoperatively those patients who could develop postoperative esophageal motor complications, the results would surely be better. This is why this study was carried out. We found alterations in the postoperative period of the vertical gastrectomy, but they were not correlated with the manometric findings in the preoperative period.

## CONCLUSION

It can not be concluded that the manometry in the preoperative period of vertical gastrectomy is a selection factor for the candidates for this operative technique.
